# Les pneumopathies aigues du nourrisson en Côte d'Ivoire: apport de la radiographie thoracique dans la recherche étiologique et la prise en charge précoce

**Published:** 2012-09-13

**Authors:** Kouamé Nágoan, Anne-Marie Nágoan-Domoua, Sétchéou Alihonou, Anhum Nicaise Konan

**Affiliations:** 1 Service de radiologie, CHU de Yopougon, 21 BP 632 Abidjan 21, Côte d'Ivoire

**Keywords:** Nourrisson, pneumopathies aigues, radiographie thoracique, Afrique, infant, acute pneumopathies, chest radiograph, Africa

## Abstract

**Introduction:**

Identifier les tableaux radio-cliniques actuels des pneumopathies aiguës du nourrisson rencontrés en Côte d'Ivoire et démontrer le rôle de la radiographie thoracique dans leur prise en charge.

**Méthodes:**

Etude rétrospective de 24 mois ayant concerné l'analyse de 165 radiographies thoraciques (RT) de face réalisées chez des nourrissons âgés de 1 à 24 mois, hospitalisés dans le service de pédiatrie du CHU de Yopougon (Abidjan-Côte d'Ivoire) pour pneumopathies aigues. Les éléments épidémio-cliniques, thérapeutiques et évolutifs ont été obtenus à partir du dossier médical des nourrissons.

**Résultats:**

L'âge moyen des nourrissons était de 9 mois avec des extrêmes entre 3 et 22 mois. Le sex-ratio était égal à 1,2. Les syndromes radiographiques étaient dominés par le syndrome alvéolaire (70,3%) suivi par l'association syndrome alvéolaire-syndrome bronchique (29,7%). Les signes radiographiques de gravité étaient présents dans 61,8%. Les entités radio-cliniques étaient représentées par les pneumopathies massives (32,7%), la pneumonie franche lobaire aigue (4,2%), les abcès du poumon (7,3%), les staphylococcies pleuro-pulmonaires (4,2%), les pleuro-pneumopathies (13,3%), le pyo-pneumothorax (4,9%), les broncho-pneumopathies (29,7%) et la primo-infection tuberculeuse (3,7%).

**Conclusion:**

A travers la mise en parallèle des entités radio-cliniques avec les éléments épidémiologiques et cliniques, la RT a permis de préjuger de l'étiologie des pneumopathies et de mettre en route immédiatement le traitement spécifique. A l'ère de la pandémie du VIH-SIDA, cette étude montre que la tuberculose pulmonaire est paradoxalement l'entité radio-clinique la plus rare.

## Introduction

Les pneumopathies aigues du nourrisson (PAN) se définissent comme une atteinte infectieuse du parenchyme pulmonaire évoluant depuis moins de 15 jours et survenant chez l'enfant entre 1 et 24 mois [1]. C'est une affection fréquente [2] en Afrique noire où elle est favorisée par les par les conditions socio-économiques et environnementales défavorables: la pauvreté, l'absence de vaccination et d'hygiène, la promiscuité et l'état nutritionnel précaire [1]. Les PAN sont extrêmement graves car responsables de morbidité et de mortalité élevées chez l'enfant [3, 4]. Elles sont le plus souvent réputées d'être d'origine bactérienne en Afrique noire [5]. Mais l'objectivation biologique de cette bactérie pose souvent problème. Elle est parfois impossible du fait de l'absence de laboratoire dans certaines régions. Lorsqu'elle est possible, le délai d'attente des résultats est plus ou moins long et l'antibiothérapie probabiliste est inappropriée sur une large proportion [6, 7]. Selon Oulai et al [6], la recherche bactériologique est rarement positive. La radiographie thoracique (RT) devient alors incontournable non seulement dans le diagnostic positif de l'atteinte infectieuse parenchymateuse mais également dans l'orientation étiologique. En effet, les signes radiographiques des PAN associés à leurs signes cliniques constituent des tableaux radio-cliniques qui permettent de présumer de l'étiologie et d'éliminer la présence d'un corps étranger dans les voies aériennes basses du nourrisson. La RT est peu onéreuse, réalisable en urgence, peu irradiante et disponible en Afrique Noire. Le but de notre travail était de répertorier et de décrire les différents tableaux radio-cliniques des PAN à Abidjan (Côte d'Ivoire) afin de permettre leur prise en charge précoce et efficace.

## Méthodes

Notre étude était rétrospective et descriptive. Elle a porté sur une période de 2 ans allant du 1er novembre 2008 au 31 Octobre 2010. Elle a concerné 165 radiographies thoraciques standard de face réalisées chez des nourrissons âgés de 1 à 24 mois. Ces enfants étaient hospitalisés dans le service de pédiatrie du centre hospitalo-universitaire (CHU) de Yopougon (Abidjan-Côte d'Ivoire) pour pneumopathies aiguës. Toutes les radiographies ont été effectuées dans le service de radiologie du même CHU. Elles ont été réalisées de face, en inspiration, en postéro-antérieure et en position débout. Un appareil de radiographie non numérisé de type os-poumons a été utilisé. La lecture des clichés radiographiques a été effectuée par des radiologues expérimentés. Elle a consisté en la recherche de corps étranger, de syndrome alvéolaire et de signes associés et ou de gravité (épanchement pleural, abcès, caverne, opacité médiastinale ou extension de l'atteinte à plusieurs lobes). Par la suite, des entités radio-cliniques étaient dégagées en fonction du contexte clinique, de la topographie de la lésion et des signes associés au syndrome alvéolaire. Les aspects épidémiologiques, cliniques, biologiques, thérapeutiques et évolutifs, obtenus à partir de la consultation des dossiers des nourrissons, nous ont permis de confirmer le diagnostic.

## Résultats

**Figure 1:**
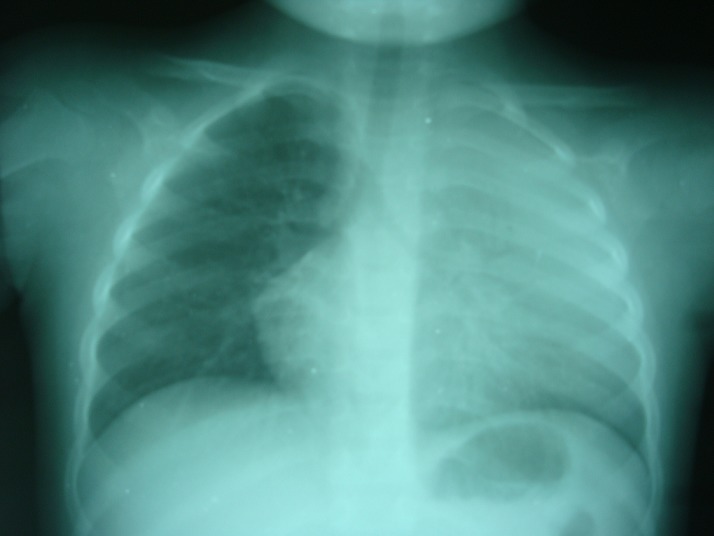
Nourrisson de 23 mois présentant un tableau clinique de toux, fièvre (40°C), dyspnée avec un syndrome de condensation pulmonaire gauche. La radiographie thoracique réalisée de face en inspiration montre un syndrome alvéolaire de tout le poumon gauche. L'aspect radio-clinique est celui d'une pneumopathie massive orientant vers l'étiologie pneumococcique

**Figure 2:**
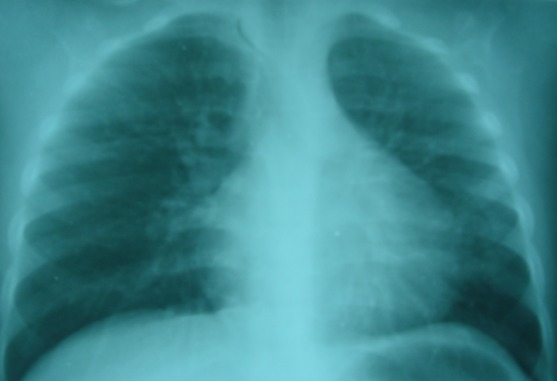
Même enfant. 10 jours après un traitement antibiotique anti-pneumococcique. La radiographie thoracique de contrôle montre une disparition totale des signes radiologiques

L'ensemble de nos résultats est résumé dans les Tableau 1, Tableau 2 et Tableau 3. L'âge moyen des nourrissons était de 9 mois avec des extrêmes entre 3 et 22 mois. Les enfants ayant une classe d'âge entre 13 et 18 mois étaient prédominants (41,2%). Le sex-ratio était égal à 1,2 (90 garçons et 76 filles). L'atteinte du poumon droit était prédominante (n = 86 soit 52,1%), suivi de l'atteinte bilatérale (n = 52 soit 31,5%) et de celle du poumon gauche (n = 27 soit16,4%). L'atteinte du lobe inferieur était prédominante (n= 52 soit 31,5%), suivi de celle d'un hémi thorax entier (n= 54 soit 32,8%), du lobe moyen (n = 33 soit 20%) et du lobe supérieur (n= 26 soit 15,7%). Les syndromes radiographiques étaient dominés par le syndrome alvéolaire (n = 116 soit 70,3%) suivi par l'association syndrome alvéolaire-syndrome bronchique (n = 49 soit 29,7%). Les signes radiographiques de gravité étaient présents dans 61,8% des cas. Ils étaient dominés par l'atteinte de plusieurs lobes à la fois (58,6%), le syndrome d'épanchement liquidien pleural (19%), le syndrome cavitaire (10,3%), le syndrome d'épanchement pleural gazeux (6,9%) et les adénopathies médiastinales (5,2%). Les tableaux radio-cliniques étaient représentés par les pneumopathies massives (Figure 1 et Figure 2) avec une proportion de 32,7%, la pneumonie franche lobaire aigue (4,2%), les abcès du poumon (7,3%), les staphylococcies pleuro-pulmonaires (4,2%), les pleuro-pneumopathies (13,3%), le pyo-pneumothorax (4,9%), les broncho-pneumopathies (29,7%) et la primo-infection tuberculeuse (3,7%).

**Tableau 1:** Répartition anatomique des pneumopathies aiguës du nourrisson

Classe d'âge (mois)	Effectif	Pourcentage
1-6	37	22,4
7-12	25	15,2
13-18	68	41,2
19-24	35	21,2
**Total**	**165**	**100**

**Tableau 2:** Signes radiographiques de gravité et ou associés au syndrome alvéolaire

Signes de gravité et ou associés	Effectif	Pourcentage
Atteinte de plusieurs lobes	54	52,9
cavitaire (abcès)	12	11,8
Epanchement pleural liquidien	22	21,6
Epanchement pleural gazeux	8	7,8
Médiastinal (adénopathie)	6	5,9
**Total**	**102**	**100**

**Tableau 3:** Tableaux radio-cliniques des pneumopathies aiguës du nourrisson

	Effectif	Pourcentage
Pneumonies franches lobaires aigues	7	4,2
Pneumonies massives	54	32,7
Abcès pulmonaire	12	7,3
Staphylococcies pleuro-pulmonaires	7	4,2
Pleuro-pneumopathies	22	13,3
Pyo-pneumothorax	8	4,9
Primo-infection tuberculeuse	6	3,7
Broncho-pneumopathies	49	29,7
**Total**	**165**	**100**

## Discussion

Les PAN intéressent le plus souvent l'enfant de sexe masculin [8, 9]. Dans notre étude le sexe ratio était légèrement en faveur des garçons (sex ratio = 1,2). Il en est de même au Togo [1] où le sex ratio est de 1,3. Selon Martinot [10], l'âge moyen de survenue de l'atteinte pleuro-pulmonaire chez l'enfant est de 9 mois. Notre étude a retrouvé des résultats similaires avec dans 41,2% des cas une prédominance de la classe d'âge entre 13 et 18 mois.

Les PAN sont responsables d'une morbidité importante chez l'enfant [8] et constituent pour cela un problème majeur de santé publique [11]. Selon Gaudelus [2], Les pneumonies de l'enfant sont fréquentes dans tous les pays et sont responsables dans les pays en voie de développement d'une mortalité non négligeable. Leur prise en charge doit, par conséquent, être faite en urgence. Mais, les PAN posent un problème de diagnostic étiologique rendant délicat voire retardant une prise en charge efficace. Selon la littérature [2], aucun signe clinique ou biologique ne permet de distinguer l'étiologie bactérienne de celle virale au cours des PAN. Et même s'il est admis qu'en Afrique, les causes bactériennes prédominent sur les étiologies virales, il est tout à fait difficile de d'isoler le germe en cause ou de le présumer sur des critères cliniques ou biologiques [12]. Tous les auteurs s'accordent sur la nécessité d'une antibiothérapie probabiliste à large spectre. Les résultats sont le plus souvent décevants avec le risque de création de souches résistantes en particulier le pneumocoque. D'autres auteurs soulignent, comme nous, la nécessité de faire appel à la radiographie pulmonaire. Au Burundi [11] le diagnostic et la prise en charge, des affections respiratoires basses des nourrissons, est basée sur des signes radio-cliniques. Sur cette base les entités radio-cliniques que Nikoyagze [11] a mis en évidence sont par ordre de fréquence les broncho-pneumopathies, les pneumopathies lobaires et les pneumopathies tuberculeuses. Au Togo [2] les broncho-pneumopathies sont également la forme radio-clinique la plus fréquente des PAN (62,5%). Dans notre étude, les broncho-pneumopathies (29,7) venaient en deuxième position après les pneumopathies massives (32,7%). Ils étaient suivis par les pleuro-pneumopathies (13,3%), les abcès du poumon (7,3%), le pyo-pneumothorax (4,9%), la pneumonie franche lobaire aigue (4,2%), les staphylococcies pleuro-pulmonaires (4,2%) et la primo-infection tuberculeuse (3,7%). Cette classification radio-clinique des PAN est basée sur des critères épidémiologiques, cliniques, biologiques et surtout sur la topographie et les signes d'accompagnement du syndrome alvéolaire radiologique. Elle a pour mérite, même si elle ne peut prétendre remplacer la bactériologie, d'orienter l'antibiothérapie probabiliste. Les implications thérapeutiques d'une telle classification apparaissent très intéressantes. Les agents responsables des pneumonies sévères ou dites massives sont *Streptococcus pneumonia* et *Hemophilus influenzae* [5]. Ces deux germes sont sensibles aux mêmes types d'antibiotiques notamment les céphalosporines de troisième génération associées aux aminosides [6]. Selon Martinot [10], l′existence d′un épanchement pleural liquidien ou gazeux ou d′images bulleuses radiologiques constituent les clefs du diagnostic de la staphylococcie pleuro-pulmonaire du nourrisson et permettent de mettre en route, sans délai, une antibiothérapie de 1^ère^ intention sur les staphylocoques méthicilino-résistants. Pour Gaudelus [2] et Sardet [13], le pneumocoque est le germe le plus souvent responsable des pleuro-pneumopathies bactériennes de l'enfant. Dans notre étude, la tuberculose pulmonaire était diagnostiquée à la radiographie sur l'atteinte pulmonaire associée à l'atteinte médiastinale ganglionnaire. Mabiala-Babela [14] confirme nos observations en démontrant que les formes intra-thoraciques de la tuberculose du nourrisson sont dominées par les broncho-pneumonies (72,6%) et les adénopathies médiastinales (40,2%). En effet quoique la tuberculose soit qualifiée comme la grande simulatrice de par ses aspects multiformes, l'association atteinte parenchymateuse et adénopathie médiastinale est évocatrice d'une primo-infection tuberculeuse. Koffi [7] à Abidjan a fait le diagnostic des pneumonies de l'enfant spécifiquement drépanocytaire sur le profil radio-clinique associé à la recherche biologique du Bacille de Koch. Sa conclusion de la faible prévalence de la tuberculose pulmonaire du nourrisson (3,7%) à l'ère du VIH-SIDA est identique à la celle de notre étude (6%). Il en est de même au Burundi [11] où la proportion de tuberculose pulmonaire parmi les pneumopathies du nourrisson est de (5%).

## Conclusion

Les PAN sont des affections fréquentes et graves constituant un problème de santé publique. La radiographie thoracique, méthode simple, peu irradiante, disponible partout en Afrique et très peu coûteuse est incontournable dans l'exploration de cette affection. La prise en charge diagnostic et thérapeutique des PAN nécessite une collaboration étroite entre le radiologue et le pédiatre. Dans notre étude, à travers la mise en parallèle des entités radio-cliniques avec les éléments épidémiologiques et cliniques, la RT a permis de préjuger de l'étiologie des pneumopathies et de mettre en route immédiatement le traitement spécifique. A l'ère de la pandémie du VIH-SIDA, cette étude démontre que la tuberculose pulmonaire est paradoxalement l'entité radio-clinique la plus rare.
